# Association of health vulnerability with adverse outcomes in older people with COVID-19: a prospective cohort study

**DOI:** 10.6061/clinics/2021/e3369

**Published:** 2021-11-23

**Authors:** Fábio Cavalcante de Assis, Michelle Cristina-Oliveira da Silva, João Carlos Geber-Júnior, Hamilton Roschel, Tiago Peçanha, Luciano Ferreira Drager, Alfredo Nicodemos Cruz Santana

**Affiliations:** I Departamento de Clinica Medica, Disciplina de Medicina de Emergencia, Faculdade de Medicina, Universidade de Brasília (UnB), Brasilia, DF, BR.; II Time de Resposta Rapida, Hospital das Clinicas HCFMUSP, Faculdade de Medicina, Universidade de Sao Paulo, Sao Paulo, SP, BR.; III Unidade de Hipertensao, Disciplina de Nefrologia, Hospital das Clinicas HCFMUSP, Faculdade de Medicina, Universidade de Sao Paulo, Sao Paulo, SP, BR.; IV Departamento de Clinica Medica, Disciplina de Clinica Geral e Propedeutica, Hospital das Clinicas HCFMUSP, Faculdade de Medicina, Universidade de Sao Paulo, Sao Paulo, SP, BR.; V Departamento de Clinica Medica, Disciplina de Reumatologia, Hospital das Clinicas HCFMUSP, Faculdade de Medicina, Universidade de Sao Paulo, Sao Paulo, SP, BR.; VI Unidade de Terapia Intensiva Cardiologica, Departamento de Cardiopneumologia, Instituto do Coracao (InCor), Faculdade de Medicina FMUSP, Universidade de Sao Paulo, Sao Paulo, SP, BR.; VII Unidade de Hipertensao, Instituto do Coracao (InCor), Faculdade de Medicina FMUSP, Universidade de Sao Paulo, Sao Paulo, SP, BR.; VIII Grupo de Pesquisa em Fisiologia Aplicada e Nutricao, Escola de Educacao Fisica e Esporte, Faculdade de Medicina FMUSP, Universidade de Sao Paulo, Sao Paulo, SP, BR.; IX Escola de Ciencias da Saude (ESCS), Brasilia, DF, BR.; X Hospital Sirio Libanes, Brasilia, DF, BR.

**Keywords:** Mortality, Triage, Respiration, Artificial, Health Vulnerability, Frail Elderly

## Abstract

**OBJECTIVES:**

Health vulnerability is associated with a higher risk of mortality and functional decline in older people in the community. However, few studies have evaluated the role of the Vulnerable Elders Survey (VES-13) in predicting clinical outcomes of hospitalized patients. In the present study, we tested the ability of the VES-13 to predict mortality and the need for invasive mechanical ventilation in older people hospitalized with coronavirus disease 2019 (COVID-19).

**METHODS:**

This prospective cohort included 91 participants aged ≥60 years who were confirmed to have COVID-19. VES-13 was applied, and the demographic, clinical, and laboratory variables were collected within 72h of hospitalization. A Poisson generalized linear regression model with robust variance was used to estimate the relative risk of death and invasive mechanical ventilation.

**RESULTS:**

Of the total number of patients, 19 (21%) died and 15 (16%) required invasive mechanical ventilation. Regarding health vulnerability, 54 (59.4%) participants were classified as non-vulnerable, 30 (33%) as vulnerable, and 7 (7.6%) as extremely vulnerable. Patients classified as extremely vulnerable and male sex were strongly and independently associated with a higher relative risk of in-hospital mortality (*p*<0.05) and need for invasive mechanical ventilation (*p*<0.05).

**CONCLUSION:**

Elderly patients classified as extremely vulnerable had more unfavorable outcomes after hospitalization for COVID-19. These data highlight the importance of identifying health vulnerabilities in this population.

## INTRODUCTION

Health vulnerability in the elderly is associated with a greater risk of functional decline and death (1,2).

Among the tools developed for this purpose, the Vulnerable Elders Survey (VES-13) stands out. It is a simple scoring system capable of identifying vulnerable elderly people in the community and includes factors such as age, self-assessed health, functional limitations, and impairments (1-3).

A score of ≥3 indicates a 4.2-fold higher risk of death and functional decline in 2 years compared with non-vulnerable elderly people (1). Additionally, for every one-point increase in the VES-13 score, the risk of death and functional decline further increases by 37% over a 5-year period (odds ratio [OR]=1.37; 95% confidence interval [CI], 1.25-1.50) (2-8). In addition, some studies have evaluated the role of VES-13 in predicting the clinical outcomes of hospitalized elderly individuals (9-12).

To date, no study has evaluated the role of VES-13 in elderly patients hospitalized for coronavirus disease 2019 (COVID-19), and there is growing interest in identifying factors that predict poor clinical evolution in elderly patients with this disease. Therefore, our objective was to assess whether health vulnerability predicts the need for invasive mechanical ventilation or mortality in this group of patients hospitalized for COVID-19 (13-22).

## METHODS

### Study population and design

This single-center, prospective cohort study was conducted at Hospital das Clínicas, Faculty of Medicine, University of São Paulo, which has been a reference institution for the treatment of patients with COVID-19 in the state of São Paulo, Brazil, since March 2020. Participants were recruited between July 2020 and December 2020. The inclusion criteria were as follows: age≥60 years, definitive diagnosis of COVID-19 after detection of severe acute respiratory syndrome coronavirus 2 (SARS-CoV-2) through real-time polymerase chain reaction using nasal and oropharyngeal swabs or positive IgG serology for SARS-CoV-2 associated with clinical and/or radiological picture compatible with COVID-19. The exclusion criteria were delirium, under exclusive palliative care, and those already on invasive mechanical ventilation. Patients were followed up until the date of death or hospital discharge. Prior to participation, eligible patients received detailed explanation of the study and provided written informed consent. The study followed the principles of the Declaration of Helsinki and was approved by the local research ethics committee (CAAE: 3351320.6.0000.0068).

### Data collection

Clinical and laboratory data were extracted from electronic medical records, and VES-13 was applied within 72h of hospital admission. Based on these data, the quick Sequential Organ Failure Assessment (qSOFA) value was calculated for all participants. We used a qSOFA cutoff of ≥2 for patients at a higher risk of an unfavorable outcome (19,22).

The VES-13 questionnaire is a scoring system with scores ranging from 0 to 10 points, consisting of 13 simple and objective questions. According to the VES-13 score, individuals were classified as non-vulnerable (0-2), vulnerable (3-7), and extremely vulnerable (1,2,8-10,12). It is noteworthy that the VES-13 was translated, adapted, and validated for the Brazilian Portuguese (23,24).

### Outcomes

The primary outcomes were mortality and need for invasive mechanical ventilation during the hospital stay.

### Statistical analysis

Initial data are expressed as absolute frequencies and percentages, mean, standard deviation, median, Q1, Q3, and minimum and maximum values. The comparison between groups for death and mechanical ventilation (yes and no) in relation to quantitative variables was performed using the Mann-Whitney test, a non-parametric technique that allows the comparison of two independent groups without any assumptions regarding data distribution (25,26). A Poisson generalized linear regression model with robust variance was used to estimate the relative risk of death and invasive mechanical ventilation (27).

A hierarchical strategy was adopted to assess the four-step insertion of variables: demographic, clinical, laboratory, qSOFA, and VES-13. The Wald test was performed to compare the nested models, and the first model was compared to a null model (28). All analyses and figures were performed using the R software version 4.0.0 (R Foundation for Statistical Computing, Vienna, Austria). A significance level of 5% (*p*<0.05) was used for all comparisons.

## RESULTS

Between July and December 2020, 165 patients were initially selected, and 91 patients met all eligibility criteria and agreed to participate. The overall characteristics of the participants are listed in Table 1.

Of the total patients, 19 (21%) died and 15 (16%) required invasive mechanical ventilation. The median and standard deviation for age were 77 (61-97) and 68 (61-91) years for patients who died and required mechanical ventilation, respectively. Systemic arterial hypertension was found in 71 (78%) and obesity in 11 (12%) patients. Regarding health vulnerability, 54 (59.4%) patients were classified as non-vulnerable, 30 (33%) as vulnerable, and 7 (7.6%) as extremely vulnerable.

**Figure 1:**
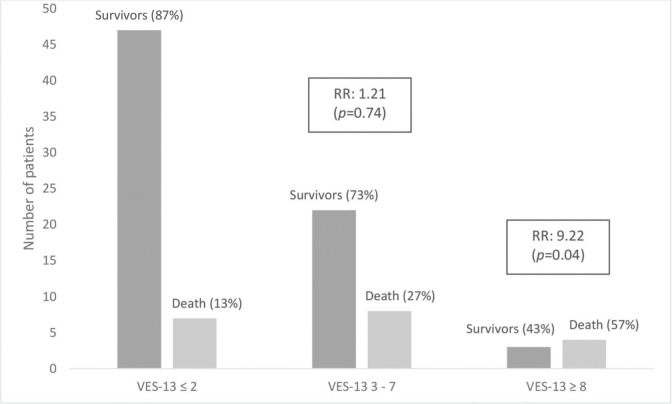
Association between VES-13 and mortality. **VES-13** = Vulnerable Elders Survey, **RR** = Relative risk.

**Figure 2:**
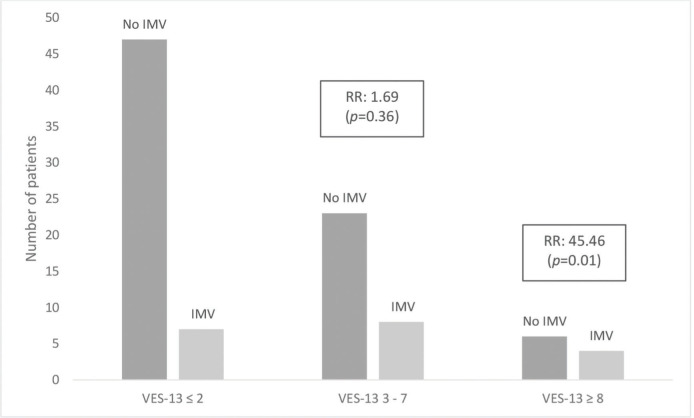
Association between VES-13 and invasive mechanical ventilation. **IMV** = invasive mechanical ventilation, **VES-13** = Vulnerable Elders Survey, **RR** = Relative risk.

Tables 2 and 3 show a hierarchical model that, after adjustments for demographic, clinical, laboratory, and qSOFA scores, revealed that patients classified as extremely vulnerable had a strong association with hospital mortality (relative risk [RR]=9.2; 95% CI 1.1-73.9; *p*<0.05) and need for invasive mechanical ventilation (RR=45; 95% CI 2.2-933; *p*<0.05). In the vulnerable group, there was a trend toward a higher risk of death and mechanical ventilation; however, the results were not statistically significant. These associations are shown in Figures 1 and 2.

Male sex was also independently associated with death (RR=4.8; 95% CI 1.5-15.4; *p*<0.01) and mechanical ventilation (RR=10.2; 95% CI 1.25-82.5; *p*<0.05). No other assessed variables, including qSOFA score of ≥2, were independently associated with the proposed outcomes. Finally, the inclusion of VES-13 in a hierarchical block model of demographic, clinical, laboratory, and qSOFA variables resulted in an improvement in the model prediction of patients requiring invasive mechanical ventilation (*p*<0.05). However, there was no improvement in the adjusted model for the patients who died (*p*=0.072).

## DISCUSSION

In this prospective cohort study, we tested the associations between health vulnerability measured by the VES-13 and clinical outcomes in elderly individuals hospitalized with COVID-19. We observed that super vulnerability was an independent predictor of death and the need for invasive mechanical ventilation during hospitalization. Thus, our findings reinforce the importance of identifying health vulnerabilities and their correlation with clinical outcomes in the elderly population. To the best of our knowledge, this is the first study to test the health vulnerability of elderly individuals hospitalized with COVID-19 using the VES-13 tool.

Recently, a prospective cohort identified that functional status prior to hospital admission, measured using the clinical frailty scale (CFS), was the only independent factor associated with the risk of death in patients aged ≥65 years and with COVID-19 during a 60-day follow-up (29). Another study involving 203 patients aged ≥75 years admitted to the emergency department with suspicion of any type of infection showed, after multivariate analysis, that the CFS score (≥5) was strongly correlated with death (OR=2.05; 95% CI 1.1-1.4; *p*<0.001) (30). These data, which correlate functional status with worse clinical outcomes, corroborate the results of our study, since functionality assessment is an essential and important part of the VES-13.

Current evidence also supports male sex as an indicator of worse prognosis. Interestingly, our results show that male sex is independently associated with death and mechanical ventilation, and this appears to be a worldwide phenomenon. An extensive meta-analysis found a similar result, although it did not exclusively assess the elderly in this study. The study, which included 46 different countries and 44 states in the United States, demonstrated that males were three times more likely to be admitted to the intensive care unit (ICU) and has a greater risk of mortality than women (31). Another study with findings corroborating our results found >50% risk of death from all causes, severe COVID-19, and ICU admission (32). Furthermore, according to the study, the risk could not be explained by patient age or comorbidities.

Differences in the innate and adaptive immune systems between men and women have been reported and may account for the female advantage in COVID-19. The female adaptive immune system has a greater number of CD4^+^ T cells, greater cytotoxic activity of CD8^+^ T cells, greater production of immunoglobulins than men, and less production of inflammatory cytokines (31,32). This difference may be related to estrogen and suggests its protective effect against the development of hyperinflammatory immune responses associated with mortality in COVID-19 (31,32).

Contrary to our analysis, a qSOFA score of ≥2 was an independent risk factor for death in patients aged ≥80 years who were infected with SARS-CoV-2 (19,22). Moreover, unlike other studies, our analysis did not identify any clinical or laboratory variables associated with the proposed outcomes (14,22).

This study has certain limitations and strengths. Despite the positive results obtained, it is important to emphasize that our study involved a single center in an underdeveloped country and the extrapolation of our results to other populations should be conducted with caution. Furthermore, our cohort had a reasonably small number of participants, which likely explains the wide CIs in our results.

Finally, some confounding factors not evaluated in our study may have influenced our results; therefore, more studies on the subject should be performed with a larger sample and involving several centers. Regarding strengths, our data were prospectively collected; therefore, the correlation between VES-13 and male sex as a causal factor of poor prognosis in elderly patients hospitalized with COVID-19 has a high degree of reliability.

## CONCLUSION

In elderly patients hospitalized with COVID-19, a final VES-13 score between 8 and 10 was associated with poor outcomes, such as death and invasive mechanical ventilation. These data highlight the importance of identifying health vulnerabilities in this population group.

## AUTHOR CONTRIBUTIONS

Assis FC, Silva MCO and Geber-Júnior JC collected the data. Assis FC, Silva MCO, Geber-Júnior JC, Drager LF, Roschel H, Peçanha T and Santana ANC planned and performed the analysis. Assis FC, Drager LF and Santana ANC contributed to the interpretation of the results. Assis FC wrote the first version of the manuscript, and other authors contributed to the improvement of the first version. Assis FC and Santana ANC revised the final version of the manuscript. Assis FC and Santana ANC supervised the project. All authors provided critical feedback and helped shape the research, analysis and manuscript.

## Figures and Tables

**Table 1:** Description of patients, characteristics, and results.

	Death		*p*-value	Invasive mechanical ventilation		*p*-value
	No (n=72)	Yes (n=19)		No (n=76)	Yes (n=15)	
Age (years)			0.0169*			0.6967
Median (Q1-Q3)	67 (63*-*76)	77 (66*-*88)		68 (63.5*-*77.5)	68 (64-78)	
Min-Max	60-89	61-97		60-97	61-91	
Creatinine			0.0719			0.0723
Median (Q1- Q3)	1.02 (0.77-1.36)	1.25 (0.92-1.8)		1.025 (0.77-1.36)	1.25 (0.92-1.81)	
Min-Max	0.34-5.25	0.67-7.41		0.34-5.25	0.82-7.41	
Lymphocytes			0.1060			0.2871
<20%	55 (75.34%)	18 (24.66%)		59 (80.82%)	14 (19.18%)	
>20%	17 (94.44%)	1 (5.56%)		17 (94.44%)	1 (5.56%)	
Sex			0.0397*			0.0038*
Male	33 (70.21%)	14 (29.79%)		34 (72.34%)	13 (27.66%)	
Female	39 (88.64%)	5 (11.36%)		42 (95.45%)	2 (4.55%)	
Ethnicity			0.5903			0.9999
Caucasian	48 (81.36%)	11 (18.64%)		27 (84.38%)	5 (15.63%)	
Non-Caucasian	24 (75%)	8 (25%)		49 (83.05%)	10 (16.95%)	
Hypertension			0.9999			0.9999
No	16 (80%)	4 (20%)		17 (85%)	3 (15%)	
Yes	56 (78.87%)	15 (21.13%)		59 (83.1%)	12 (16.9%)	
Obesity			0.4474			0.6839
No	62 (77.5%)	18 (22.5%)		66 (82.5%)	14 (17.5%)	
Yes	10 (90.91%)	1 (9.09%)		10 (90.91%)	1 (9.09%)	
Smoking			0.4799			0.6951
No	62 (80.52%)	15 (19.48%)		65 (84.42%)	12 (15.58%)	
Yes	10 (71.43%)	4 (28.57%)		11 (78.57%)	3 (21.43%)	
qSOFA			0.2321			0.2001
0/1	65 (81.25%)	15 (18.75%)		65 (81.25%)	15 (18.75%)	
2/3	7 (63.64%)	4 (36.36%)		11 (100%)	0 (0%)	
VES-13			0.0172*			0.4108
0-2	47 (87.04%)	7 (12.96%)		47 (87.04%)	7 (12.96%)	
3-7	22 (73.33%)	8 (26.67%)		23 (76.67%)	7 (23.33%)	
8-10	3 (42.86%)	4 (57.14%)		6 (85.71%)	1 (14.29%)	

**VES-13** = Vulnerable Elders Survey. **qSOFA** = quick Sequential Organ Failure Assessment Score.

* *p*<0.05.

**Table 2:** Hierarchical model of variables in relation to mortality.

Relative risk of death (n=91)																
	Model 1				Model 2				Model 3				Model 4			
	RR	95% CI		*p-*value	RR	95% CI		*p*-value	RR	95% CI		*p*-value	RR	95% CI		*p*-value
Age (years)	1.0580	1.0159	1.1020	0.0065	1.0588	1.0105	1.1093	0.0164	1.0521	1.0032	1.1033	0.0363	1.0259	0.9681	1.0871	0.3880
Male sex	2.6133	1.1164	6.1173	0.0269	1.9732	0.8517	4.5715	0.1128	2.3544	1.1478	4.8295	0.0195	4.8197	1.5059	15.4255	0.0081*
Caucasian ethnicity	1.4026	0.6240	3.1530	0.4129	1.3710	0.5676	3.3118	0.4832	1.3602	0.5563	3.3262	0.5001	1.3897	0.6061	3.1863	0.4369
Hypertension					0.9056	0.3514	2.3336	0.8373	0.8769	0.3575	2.1510	0.7741	0.9295	0.3713	2.3274	0.8760
Obesity					0.5122	0.0778	3.3700	0.4864	0.5670	0.0779	4.1254	0.5752	0.4560	0.1012	2.0551	0.3066
Smoking					2.0294	0.7233	5.6938	0.1787	2.1937	0.7767	6.1957	0.1381	2.2826	0.8012	6.5032	0.1223
Lymphocytes<20%					3.9566	0.9884	15.8380	0.0520	4.3618	0.9849	19.3179	0.0524	5.7082	0.6512	50.0386	0.1158
Creatinine					1.1655	0.9130	1.4879	0.2190	1.1709	0.9124	1.5026	0.2151	1.1051	0.8333	1.4657	0.4878
qSOFA (2/3 *versus* 0/1)									2.0826	1.0384	4.1768	0.0388	0.9916	0.3126	3.1451	0.9885
VES-13 (3-7 *versus* 0-2)													1.2053	0.3999	3.6330	0.7401
VES-13 (8-10 *versus* 0-2)													9.2154	1.1480	73.9774	0.0366*
Wald test for change	<0.0001				0.1927				0.0420				0.0721			

* *p*<0.05. **VES-13 =** Vulnerable Elders Survey, **qSOFA =** quick Sequential Organ Failure Assessment Score, **RR** = Relative risk.

**Table 3:** Hierarchical model of variables in relation to the need for invasive mechanical ventilation.

Relative Risk of Invasive Mechanical Ventilation (n=91)																
	Model 1				Model 2				Model 3				Model 4			
	RR	95% CI		*p-*value	RR	95% CI		*p*-value	RR	95% CI		*p*-value	RR	95% CI		*p*-value
Age (years)	1.0028	0.9491	1.0595	0.9213	1.0008	0.9347	1.0716	0.9815	1.0021	0.9327	1.0767	0.9544	0.9714	0.9114	1.0352	0.3714
Male sex	6.1254	1.4266	26.3008	0.0148	5.0148	1.1456	21.9520	0.0323	4.8872	0.9732	24.5425	0.0540	10.1919	1.2589	82.5111	0.0296*
Caucasian ethnicity	1.0983	0.4257	2.8336	0.8462	1.1617	0.4148	3.2539	0.7755	1.1760	0.4448	3.1090	0.7438	0.9589	0.3655	2.5158	0.9320
Hypertension					1.1557	0.3749	3.5620	0.8011	1.2240	0.4110	3.6455	0.7166	1.1417	0.3728	3.4968	0.8165
Obesity					0.5412	0.0506	5.7826	0.6114	0.5152	0.0498	5.3289	0.5780	0.4411	0.0918	2.1185	0.3067
Smoking					1.5004	0.5031	4.4749	0.4668	1.3585	0.4853	3.8033	0.5597	1.5129	0.5656	4.0468	0.4095
Lymphocytes<20%					2.2561	0.3929	12.9540	0.3615	1.7011	0.2818	10.2695	0.5625	1.3388	0.2441	7.3433	0.7369
Creatinine					1.1665	0.9093	1.4966	0.2256	1.1494	0.9073	1.4561	0.2487	1.1355	0.9480	1.3602	0.1676
qSOFA (2/3 *versus* 0/1)									**	**	**	**	**	**	**	**
VES-13 (3-7 *versus* 0-2)													1.6895	0.5523	5.1687	0.3580
VES-13 (8-10 *versus* 0-2)													45.4551	2.2123	933.9649	0.0133*
Wald test for change	0.0566				0.5593				<0.0001				0.0491*			

* *p*<0.05. **It was not possible to estimate because it showed a Quasi-complete separation. **VES-13** = Vulnerable Elders Survey. **qSOFA** = quick Sequential Organ Failure Assessment Score. **IMV =** invasive mechanical ventilation, **RR** = Relative risk.
